# Correction to “Genome‐wide pleiotropy analysis of longitudinal blood pressure and harmonized cognitive performance measures”

**DOI:** 10.1002/alz.70806

**Published:** 2025-10-08

Kang M, Ang TFA, Devine SA, et al. Genome‐wide pleiotropy analysis of longitudinal blood pressure and harmonized cognitive performance measures. *Alzheimer's Dement*. 2025; 21:e70681. doi:10.1002/alz.70681

In this paper, Moonil Kang's affiliation is listed incompletely. In addition to affiliation no. 1, “Department of Medicine (Biomedical Genetics), Boston University Chobanian & Avedisian School of Medicine, Boston, Massachusetts, USA,” the author should have a current affiliation, “College of Pharmacy, Sookmyung Women's University, Seoul, Republic of Korea,” as a second affiliation.

We apologize for this error.

